# The Impact of Sleep Interventions on Athletic Performance: A Systematic Review

**DOI:** 10.1186/s40798-023-00599-z

**Published:** 2023-07-18

**Authors:** Lúcio A. Cunha, Júlio A. Costa, Elisa A. Marques, João Brito, Michele Lastella, Pedro Figueiredo

**Affiliations:** 1 Research Center in Sports Sciences, Health Sciences and Human Development, CIDESD, University of Maia, Maia, Portugal; 2 Portugal Football School, Portuguese Football Federation, Cruz Quebrada, Portugal; 3 School of Sport, Exercise and Health Sciences, Loughborough University, Loughborough, UK; 4 Appleton Institute for Behavioural Science, Central Queensland University, Adelaide, Australia; 5 Physical Education Department, College of Education, United Arab Emirates University, Al Ain, Abu Dhabi, UAE; 6 Research Center in Sports Sciences, Health Sciences and Human Development, CIDESD, Vila Real, Portugal

**Keywords:** Sleep interventions, Athletes, Performance

## Abstract

**Background:**

Sleep is essential for maximal performance in the athletic population. Despite that, the sport context has many factors that can negatively influence athletes’ sleep and subsequent recovery.

**Objectives:**

The purpose of this systematic review was to synthesize the most recent literature regarding sleep interventions aimed at improving sleep and subsequent performance in athletes.

**Methods:**

The present systematic review was conducted based on the PRISMA guidelines and the PICOS approach. The search was conducted in May 2022 using the electronic database PubMed, SPORTDiscus via EBSCOhost, and Web of Science. Once extracted, studies were included if they met the following criteria: (1) participants were athletes of individual or team sports; (2) implemented an intervention aimed at improving sleep; (3) measured at least one objective performance/recovery outcome; and (4) reported the relationship between sleep and performance.

**Results:**

The search returned 1584 records. Following the screening, a total of 25 studies met our inclusion criteria. All the included articles were intervention studies published between 2011 and 2021. The included studies implemented various sleep interventions, such as sleep hygiene, naps, sleep extension, light manipulation, cold water immersion, mindfulness, or a combination of two or more strategies. Sleep extension and naps were the most representative and most effective strategies to improve sleep and performance. Mindfulness and light manipulation demonstrated promising results, but more studies are needed to confirm these findings. Sleep hygiene, removing electronic devices at night, and cold water immersion had no effects on sleep and subsequent performance/recovery, but these results are based on a few studies only.

**Conclusion:**

While acknowledging the limited amount of high-quality evidence reviewed, it appears that increasing sleep duration at night or through napping was the most effective interventions to improve physical and/or cognitive performance.

*Protocol Registration* This protocol was registered in the International Platform of Registered Systematic Review and Meta-Analysis Protocols (INPLASY) on May 11, 2022, with the registration number INPLASY202250069.

## Key Points

- Increasing sleep duration through naps or night-time sleep may positively impact physical and/or cognitive performance.
- Extending sleep duration by 46–113 min in athletes that habitually sleep ~ 7 h per night may be a general recommendation for future sleep extension programs.
- Supplementing sleep during the day with a 20–90-min nap can improve performance outcomes after a regular night and restore performance decrements to baseline levels after a night with partial sleep restriction.

## Introduction

Sleep is a biological need crucial for human health and well-being [1]. During periods of sleep restriction or deprivation, general health is negatively affected, particularly the immune system, endocrine, physical, or cognitive functions [2–4]. While the general sleep recommendations advocate that adults obtain 7–9 h of sleep per night to maintain optimal health and functioning [5], many adults do not comply with these recommendations. For athletes, often exposed to high-intensity training, it is recognized that sleep is the most important method for psychological and physiological recovery [6, 7]. Elite athletes report needing approximately 8 h of sleep per night to feel rested [8]. However, elite athletes often sleep less than 7 h [8, 9] due to several potential sport and non-sport-related factors, which may vary across sports. Several sport-specific factors are associated with sleep inadequacy, including transmeridian travel (disrupting the circadian rhythm and exposing the athletes to an unfamiliar sleeping environment), cognitive arousal on the night before a competition, evening competitions, high training loads, and early morning training [10, 11]. In addition, various non-sport factors, such as social demands, work/study commitments (e.g., sponsorships), lifestyle choices (e.g., diet), individual characteristics (e.g., age), attitudes and beliefs (e.g., societal influence), and family commitments, have been linked with inadequate sleep in athletes [10]. Despite some mixed results, a decline in physical and cognitive performance can occur after a night of sleep restriction [4, 12, 13]. For example, two studies with runners and judo athletes showed that after a night with partial sleep restriction (4 h of sleep), endurance performance, muscle strength, and power were negatively affected [14, 15]. A night with partial sleep restriction can also affect the execution of motor skills that require a high cognitive dimension, such as reported for handball goalkeepers [16], dart players [17], and tennis players [18].

Given the growing concern about athletes' sleep, studies examining sleep interventions have grown exponentially over the recent years [19]. To date, studies examining the sleep/wake behavior of athletes have focused on nutrition [13, 20], sleep hygiene [21], and sleep extension/napping [22, 23]. In a systematic literature review evaluating the effectiveness of sleep interventions for athletes specifically aimed at improving performance outcomes, Bonnar et al. [24] showed that sleep extension was the most beneficial intervention, while napping, sleep hygiene, and post-exercise recovery strategies provided mixed results. However, this review was conducted over 5 years ago. Since then, many studies have been published, and our understanding of the impact of sleep interventions on athletic performance has improved. More recently, in an expert consensus about athletes’ sleep, Walsh et al. [10] narratively reviewed the studies of the general phenomena of athlete’s sleep. Despite being very comprehensive, the study included only a few reports regarding sleep interventions that explored the effect of sleep extension, sleep hygiene and naps on athletic performance. In this regard, a more comprehensive review of the literature on the entire spectrum of sleep interventions is needed.

Therefore, this systematic review aimed to update and expand the findings of Bonnar et al. [24] and synthesize the most recent literature regarding sleep interventions aimed at improving sleep and subsequent performance in athletes.

## Methods

### Protocol and Registration

This protocol was registered in the International Platform of Registered Systematic Review and Meta-Analysis Protocols (INPLASY) on May 11, 2022, with the registration number INPLASY202250069.

### Eligibility Criteria

This systematic review was conducted based on the PRISMA guidelines [25, 26] and the PICO approach [27]. The PICO approach was established as follows: Population: individual or team sports athletes; Intervention: strategies to improve or extend sleep; Comparators: control group or a baseline phase without sleep intervention; Outcomes: subjective and/or objective measurement of sleep and physical and/or cognitive performance. No sex or age restriction was applied. To be considered for the first screening, the studies had to be published or in-press in peer-reviewed journals (i.e., abstracts published in conference proceedings, books, theses, and dissertations were not considered), published in English, and have an abstract available for screening.

### Search Strategy

The articles were searched in May 2022, and three electronic databases were used: PubMed, SPORTDiscus via EBSCOhost, and Web of Science. In each database, the following descriptors covering the characteristic of participants, intervention, and outcomes were used: (athlet* OR sport*) AND (sleep AND (education OR hygiene OR Duration OR extension OR therapy OR strateg*) OR nap*) AND (performance OR competiti* OR recovery OR fatigue OR outcome*). Searches were conducted using ‘‘title and abstract’’ (PubMed), ‘‘topic’’ (Web of Science), and “abstract” (SPORTDiscus via EBSCOhost). Discrepancies in the search fields are due to differences in the options available in the databases. Filters for “English” and “articles” were applied. A reference management software (EndNote 20, Clarivate Analytics, USA) was used to import and analyze all references.

### Study Selection

Studies were included if they met the following criteria: (1) participants were athletes of individual or team sports, from trained to world-class athletes [28]; (2) implemented an intervention aimed at improving sleep; (3) measured at least one objective performance/recovery outcome; and (4) reported the relationship between sleep and performance.

Studies on referees or military tactical athletes, studies reporting only subjective performance measures, and interventions that included sleep medication were excluded.

### Data Collection Process

**Fig. 1:**
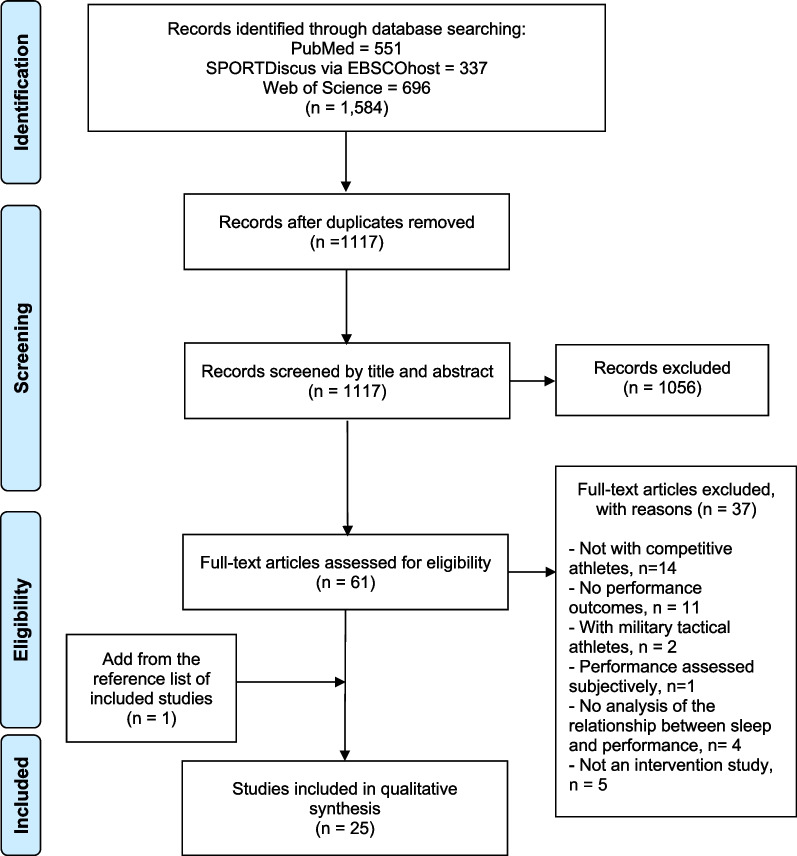
Flowchart of the systematic review following the PRISMA statement

Before starting the article selection process, duplicate citations obtained from the different databases were eliminated. Then, two evaluators (LC and JC) independently examined the article title, abstract, and keywords in the first screening stage according to the established inclusion and exclusion criteria. Subsequently, full-text articles of those citations were read and selected if suitable for inclusion. In cases of disagreement, a third reviewer (PF) was consulted. The detailed data collection process is shown in a flowchart (Fig. 1).

### Data Items

For the papers included in the analysis, we considered information about: (1) geographical location where the study was conducted; (2) study design; (3) sport; (4) sample (size, age, and sex); (5) type of intervention (e.g., sleep hygiene, napping, or sleep extension); (6) sleep assessment (e.g., questionnaires, actigraphy, or polysomnography; (7) performance/recovery assessment test and related physiological outcomes (e.g., psychomotor vigilance task, countermovement jump, or Wingate test; creatine kinase, heart rate variability (HRV), or lactate); and (8) main results.

### Risk-of-Bias Assessment

We applied the revised Cochrane risk-of-bias tool for randomized trials (RoB2) [29, 30] to assess the potential risk of bias. RoB2 is an outcome-focused, domain-based tool that evaluates the risk of bias in outcomes in individually randomized, parallel-group trials, randomized crossover trials (RCT), and cluster RCTs [29, 30]. RoB2 has five risk-of-bias domains covering the different aspects of the trial design, conduct, and reporting. These include: (1) bias arising from the randomization process; (2) bias due to deviations from intended interventions; (3) bias due to missing outcome data; (4) bias in the measurement of the outcome; and (5) bias in the selection of the reported results. We also applied the RoB2 version for crossover trials, which considers the within-participants design not addressed by the RoB2 for randomized trials and includes an additional domain: bias arising from period and carryover effects and an additional question on domain “bias in the selection of the reported results.” In both RoB2 and RoB2 for crossover trials, responses to signaling questions are mapped using a decision algorithm to determine each risk-of-bias domain judgment [30]. Based on the domain-level assessment, the overall risk-of-bias judgment was made for each assessed outcome in each trial.

The risk-of-bias assessment tool for non-randomized studies (RoBANS) [31] was applied to analyze non-randomized studies included. The RoBANS is a domain-based evaluation tool, compatible with the Cochrane risk-of-bias tool [31], that has six risk-of-bias domains: (1) selection of participants; (2) confounding variables; (3) measurement of exposure; (4) blinding of outcome assessments; (5) incomplete outcome data; and (6) selective outcome reporting. Two researchers (LC and JC) independently applied both tools (RoB2 and RoBANS). After completion, the three tables were compared, and all disagreements were discussed and reanalyzed until consensus was achieved.

## Results

### Study Selection

The search strategy returned 1584 records. Of the 61 studies retained for full-text screening, we excluded 37 studies that did not meet the inclusion criteria (Fig. 1). An additional article identified through the included studies' reference lists was included. Twenty-five studies were therefore eligible for review.

### Characteristics of Included Studies

The characteristics of the included studies are presented in Table 1.

**Table 1:** Study characteristics of included articles

Study, country	Study design	Sport	Sample (size, age*, sex)	Intervention	Sleep assessment	Performance/recovery assessment test	Main results
*Sleep hygiene*							
Fullagar et al. [54] Germany	Randomized crossover trial No. of groups: two	Soccer	20, NR, 100% male	Sleep hygiene after friendly match with a kick-off time of 20:45 versus no sleep hygiene	Actigraphy and sleep diaries	REC: The Yo–Yo intermittent recovery test—level 2 and CMJ 12 h and 36 h post-match, submaximal interval-based running test 18 and 42 h post-match with a heart rate interval monitoring system; c-reactive protein, creatine kinase, and urea 10, 20, 34, and 44 h post-match	No differences in recovery of exercise performance or blood markers; sleep hygiene $\uparrow$ total sleep time and number wake episodes compared with no sleep hygiene;
Van Ryswyk et al. [48] Australia	Pre–post-trial No. of groups: one	Australian football	25, 23.7 ± 2.0 y, 100% male	Sleep hygiene	Actigraphy, questionnaire (Pittsburgh Sleep Quality Index), and sleep diaries	CP: psychomotor vigilance task	No differences in CP or objective sleep parameters
*Napping*							
Boukhris et al. [35] Tunisia	Randomized crossover trial No. of groups: three	Soccer, rugby, and handball	14, 20.3 ± 3.0 y, 100% male	40-min nap versus 90-min nap versus no nap	Nap: questionnaire (nap quality using a scale ranging from 0 to 10) Night prior experiment: actigraphy	CP: DCT PP: knee extensors maximal voluntary isometric contraction and 5-m SRT	40- and 90-min nap: $\uparrow$ attention (DCT), KEMIVC, and 5mSRT; 40-min nap versus 90-min nap:↑ DCT, KEMIVC, and ↑ total distance in 5-m SRT (favoring 90-min nap)
Boukhris et al. [39] Tunisia	Randomized crossover trial No. of groups: two	Soccer, rugby, and handball	14, 20.3 ± 3.0 y, 100% male	40-min nap versus no nap	Nap: questionnaire (nap quality using a scale ranging from 0 to 10) Night prior experiment: actigraphy	REC: CK, LDH, ASAT, ALAT, and CRP PP: 5-m SRT	Nap $\uparrow$ recovery and 5-m SRT performance (total and highest distance)
Daaloul et al. [49] Tunisia	Randomized crossover trial No. of groups: two	Karate	13, 23.0 ± 2.0 y, 100% male	30-min nap versus no nap, after a reference night and after a night with sleep restriction	Actigraphy and sleep diaries	CP: SRT, MRT, and LRT PP: SJ, CMJ, and karate-specific test	After reference night: Nap $\uparrow$ SRT, MRT, and LRT; no differences in physical performance After a PSD night: $\downarrow$ MRT, LRT, CMJ, and KST, no differences in SRT and SJ; Nap restored performance decreases in SRT, MRT, and LRT
Suppiah et al. [52] Singapore	Studies 1 and 2: randomized crossover trial No. of groups: two	Study 1: shooters Study 2: track and field	Study 1: 12, 13.8 ± 1.0 y, 100% male Study 2: 19, 14.8 ± 1.1 y, 100% male	30-min nap versus no nap	Nap: wireless dry electroencephalographic sensor Night prior experiment: actigraphy	Study 1: PP: shooting assessment (20 competition shots) and HRV Study 2: PP: 20-m sprint	Study 1: no differences in shooting performance or HRV after a nap Study 2: $\downarrow$ 20-m sprint performance
Petit et al. [36] France	Counterbalanced and randomized crossover trial No. of groups: two	From different sports	16, 22.2 ± 1.7 y, 100% female	20-min nap versus no nap in normal conditions and at 5-h advanced sleep schedules (simulated jet lag)	Polysomnography	PP: Wingate test and plasma lactate	No differences in physical performance and plasma lactate under normal conditions or simulated jet lag
Romdhani et al. [45] Tunisia	Counterbalanced and randomized crossover trial No. of groups: one	Judo	9, 18.5 ± 0.9 y, 100% male	20-min nap versus 90-min nap versus no nap after a night with sleep restriction	Nap subjective quality	CP: multiple-choice reaction time and simple reaction time (using a specialized software) PP: RAST	N20 versus no nap: $\uparrow$ in MCRT, $\uparrow$ maximum power in RAST; no difference in SRT performance N90 versus no nap: no differences in MCRT or SRT performance; $\uparrow$ maximum, minimum, and mean power and fatigue index in RAST
Morita et al. [42] Japan	Non-RCT No. of groups: two	Softball	16, IG = 22.1 ± 0.8 y, CG = 22.0 ± 0.9 y, 100% female	2-h nap versus no nap	Actigraphy, sleep diaries, and polysomnography	CP: three-ball cascade juggling	IG: $\uparrow$ Three-ball cascade juggling performance CG: = Three-ball cascade juggling performance
Hsouna et al. [40] Non-reported	Counterbalanced crossover trial No. of groups: two	Soccer	12, 23 ± 3 y, 100% male	40-min nap versus no nap after a simulated soccer match on previous night (kick-off at 21:00)	Nap: sleepiness (Stanford Sleepiness Scale) upon waking Night prior experiment: actigraphy	PP: 5-m SRT	$\uparrow$ total and best distance after nap; no differences in fatigue index
Nishida et al. [44] Japan	Randomized crossover trial No. of groups: three	Handball	11, 20.7 ± 1.2 y, 100% male	60-min nap versus 20-min nap versus no nap	Nap: subjective quality (visual analog scale, 0–10) and sleepiness before and after (Karolinska Sleepiness Scale) Night prior experiment: wearable device (Fitbit charge 3) Sleep quality: questionnaire (Pittsburgh Sleep Quality Index)	REC: HRV during nap PP: Handball performance (20-m turnaround run, 10-m load run, and ball shooting)	No differences in handball performance or HRV; 60-min nap $\uparrow$ shooting accuracy in individuals with poor sleep quality; HRV correlated negatively with 10-m load run in 60-min nap
*Sleep extension*							
Roberts et al. [37] Australia	Counterbalanced crossover trial No. of groups: one	Cycling and triathlon	9, 30 ± 6 y, 100% male	Three consecutive days of sleep extension (30% more than a normal night) versus sleep restriction (30% less than a normal night) versus normal night	Actigraphy and sleep diaries	CP: psychomotor vigilance task PP: ETT	Sleep extension compared with NN: $\uparrow$ PVT and ETT performance; SR compared with NN: $\downarrow$ PVT and ETT performance
Schwartz and Simon [43] USA	Pre–post-trial No. of groups: one	Tennis	12, 20.2 y (18–22),42% male	7 days of sleep extension (at least 9 h), including naps	Sleep diaries	PP: serving accuracy	$\uparrow$ Serving accuracy and TST
Swinbourne et al. [50] New Zealand	Pre–post-trial No. of groups: one	Rugby	25, 25 ± 2.7 y, 100% male	Sleep extension (at least 10 h of sleep) via sleep scheduling advice, strategic napping, and sleep hygiene versus control	Actigraphy, questionnaire (Pittsburgh Sleep Quality Index), and sleep diaries	CP: the five-minute psychomotor vigilance test	$\uparrow$ reaction time performance, $\uparrow$ total sleep time, and $\uparrow$ sleep efficiency compared to control
Mah et al. [41] USA	Pre–post-trial No. of groups: one	Basketball	11, 19.4 ± 1.4 y, 100% male	Sleep extension (at least 10 h in bed per night, with a regular sleep–wake schedule and napping encouraged)	Actigraphy and sleep diaries	CP: psychomotor vigilance task PP: specific to basketball (timed 282 feet sprint, free throw, and three-point shooting accuracy)	$\uparrow$ Psychomotor vigilance performance, timed 282 feet sprint, free throw, three-point shooting accuracy, and TST
*Remove electronic devices*							
Jones et al. [34] Australia	RCT No. of groups: two	Study 1: water polo Study 2: Triathlon	Study 1: 13, 17 ± 1 y, 46% male Study 2: 12, 17 ± 1 y, 67% male	Remove electronic devices in the evening versus unrestricted electronic device use	Actigraphy and sleep quality diaries	Study 1: none Study 2: CP: psychomotor vigilance test	Study 2: no differences in cognitive performance or sleep
Dunican et al. [32] Australia	Non-RCT No. of groups: two	Judo	18, 18 ± 2 y, 56% male	Electronic devices removed for a 48-h period versus unrestricted electronic device use	Actigraphy and sleep diaries	CP: The Cogstate research tool PP: The single leg three hop test	No differences between groups in cognitive or athletic performance and sleep quality and quantity
*Light interventions*							
Zhao et al. [38] China	RCT No. of groups: two	Basketball	20, 18.6 ± 3.6 y, 100% male	30-min red-light therapy every night versus no light intervention (placebo group) with the same device (device off)	Questionnaire (Pittsburgh Sleep Quality Index) and serum melatonin	PP: Cooper 12-minute run	Red-light therapy group: $\uparrow$ distance in Cooper 12-min run performances, $\uparrow$ serum melatonin, and $\uparrow$ sleep quality Placebo group: no differences in Cooper 12-min run test performance, serum melatonin, or sleep
Rosa et al. [51] Brazil	Pre–post-trial No. of groups: one	Swimming	22, 24.8 ± 3.4 y, 50% male	Bright-light therapy (with portable glasses) and sleep hygiene	Actigraphy and sleep diaries	CP: psychomotor vigilance test	$\uparrow$ reaction time performance, delayed sleep/wake cycles, and $\downarrow$ total sleep time
*Mindfulness*							
Jones et al. [33] USA	Non-RCT No. of groups: two	Rowing	27, 18–23 y, 100% female	Mindfulness-based stress reduction versus no intervention	Actigraphy and questionnaire (Pittsburgh Sleep Quality Index and Epworth Sleepiness Scale)	PP: rowing performance (6000 m ergometer test)	$\uparrow$ in rowing performance and subjective sleep quality, and $\downarrow$ in sleepiness, compared with control group
*Cold water immersion*							
Chauvineau et al. [55] France	Counterbalanced and randomized crossover trial No. of groups: three	Running	12, 28 ± 5.8 y, 100% male	Whole CWI versus partial CWI versus control (sitting for 10 min)	Actigraphy, questionnaire (the Spiegel Sleep Inventory), polysomnography, and core body temperature	REC: Hooper Index and creatine kinase pre- and post-simulated trial, post-CWI, 24 h and 48 h; the Total of Recovery Scale post-CWI, 24 h and 48 h; CMJ and KEMIVC pre- and post-simulated trial, 24 h and 48 h; Nocturnal HRV in simulated trial day	No differences in fatigue or muscle damage, core temperature, TST, SE, WASO, and latency; CWI $\downarrow$ arousals; whole CWI $\downarrow$ parasympathetic modulation
*Combination of two or more strategies*							
Duffield et al. [56] Australia	Randomized crossover trial No. of groups: two	Tennis	8, 20.9 ± 3.6 y, 100% male	Sleep hygiene, CWI, and full-body compression versus control (passive stretching)	Actigraphy	REC: fatigue, vigor (the Brunel Mood Scale), muscle and joint soreness (1–10 Likert scale)	$\downarrow$ muscle and joint soreness, no differences in vigor; large effect sizes though non-significant results for $\downarrow$ fatigue, and $\uparrow$ TST; no differences in SE or SL
Lever et al. [53] Australia	Randomized crossover trial No. of groups: two	Tennis	17, 15.7 ± 1.1 y, 58% male	Sleep hygiene and mindfulness versus no intervention	Actigraphy and sleep diaries	PP: match performance (games won or lost)	No differences in match performance; $\uparrow$ TST, no change in SE, SL, WASO, WE, and WED
Romdhani et al. [47] Tunisia	Counterbalanced and randomized crossover trial No. of groups: four	Judo	23, 19.78 ± 1.41 y Study 1: 9, 100% male Study 2: 14, 100% male	Study 1: after a night with sleep restriction, no nap with placebo (NNP) versus no nap with caffeine (NNC) versus 20-min nap with placebo (NP) versus 20-min nap with caffeine (NC) Study 2: after a normal night, NNP versus NNC versus NP versus NC	Nap subjective quality (100-mm analog scale) and sleepiness before and after (Epworth sleepiness scale)	CP: simple and two-choice reaction time (using a specialized software)	Study 1: $\uparrow$ SRT performance in NNC; $\uparrow$ TCRT performance in NP and NC; $\downarrow$ sleepiness in NNC, NP, and NC Study 2: $\uparrow$ SRT performance in NNC; $\uparrow$ TCRT performance in NP and NC; $\downarrow$ sleepiness in NP
Romdhani et al. [46] Tunisia	Counterbalanced and randomized crossover trial No. of groups: five	Judo	9, 18.78 ± 1.09 y, 100% male	Normal sleep night without nap (NSN) versus NNP versus NNC versus 20-min NP versus 20-min NC, after a night with sleep restriction	Nap subjective quality (100-mm analog scale)	PP: RAST and plasma lactate	20-min NC $\uparrow$ RAST performance, compared with NNP, NNC, and 20-min NP; $\downarrow$ RAST performance in NNP and NNC, compared with NSN; NC and NP restored RAST performance compared with NSN; 20-min NP $\uparrow$ Pmax compared with NNP; lactate $\uparrow$ with 20-min NC and NP, compared with NNP

*CG* Control group, *CP* Cognitive performance, *PP* Physical performance, *REC* Recovery, *IG* Intervention group, *NR* Not reported, *RCT* Randomized controlled trial, *CK* Creatine kinase, *LDH* Lactate dehydrogenase, *ASAT* Aspartate aminotransferase, *ALAT* Alanine aminotransferase, *CRP* C-reactive protein, *DCT* Digit cancelation test, *5-m SRT* 5-m shuttle run test, *KEMIVC* Knee extensors maximal isometric voluntary contraction, *SRT* Simple reaction time, *MRT* Mental rotation test, *LRT* Lower reaction test, *SJ* Squat jump, *TST* Total sleep time, *SE* Sleep efficiency, *SL* Sleep latency, *WASO* Wake after sleep onset, *WE* Wake episodes, *WED* Wake-episode duration, *SE* Sleep efficiency, *SR* Sleep restriction, *NN* Normal night, *PSR* Partial sleep restriction, *ETT* Endurance time trial, *PVT* Psychomotor vigilance task, *HRV* Heart rate variability, *HR* Heart rate, *RAST* Running-based anaerobic sprint test, *CWI* Cold water immersion

*Age is presented as mean ± standard deviation or range

All the included articles were intervention studies published between 2011 and 2021 (60% crossover trials, 8% RCT, and 32% non-RCT). The number of participants in the included studies ranged from 9 to 31 athletes, with an age range from 13 to 33 years. Studies covered 17 different sports (11 studies included team sports, 13 included individual sports, and 1 was unreported). Of the 25 studies analyzed, 17 included only male athletes, 3 had only female athletes, and 5 included both male and female athletes. Finally, the level/category of the athletes was highly heterogeneous and represented mainly by just one or two studies, except for trained athletes that were included in nine studies [32–40], highly trained athletes in four studies [41–44], and elite athletes in three studies [45–47].

### Risk of Bias

**Fig. 2:**
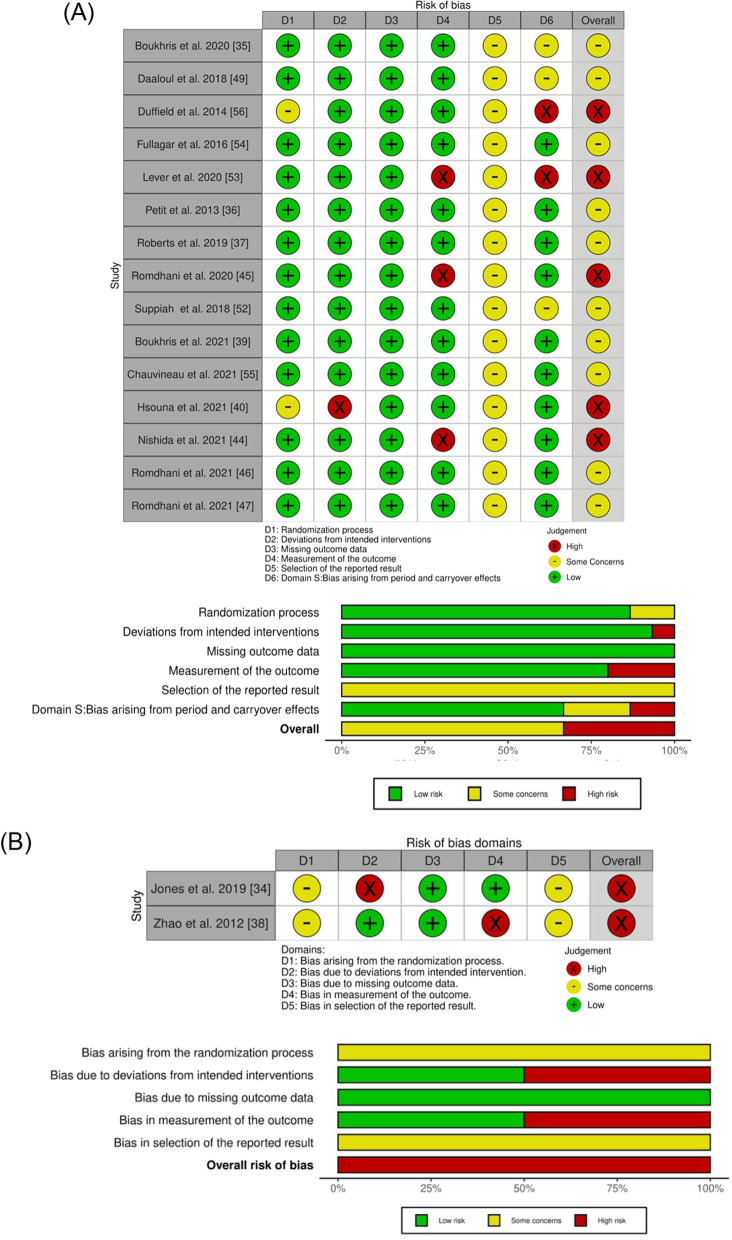

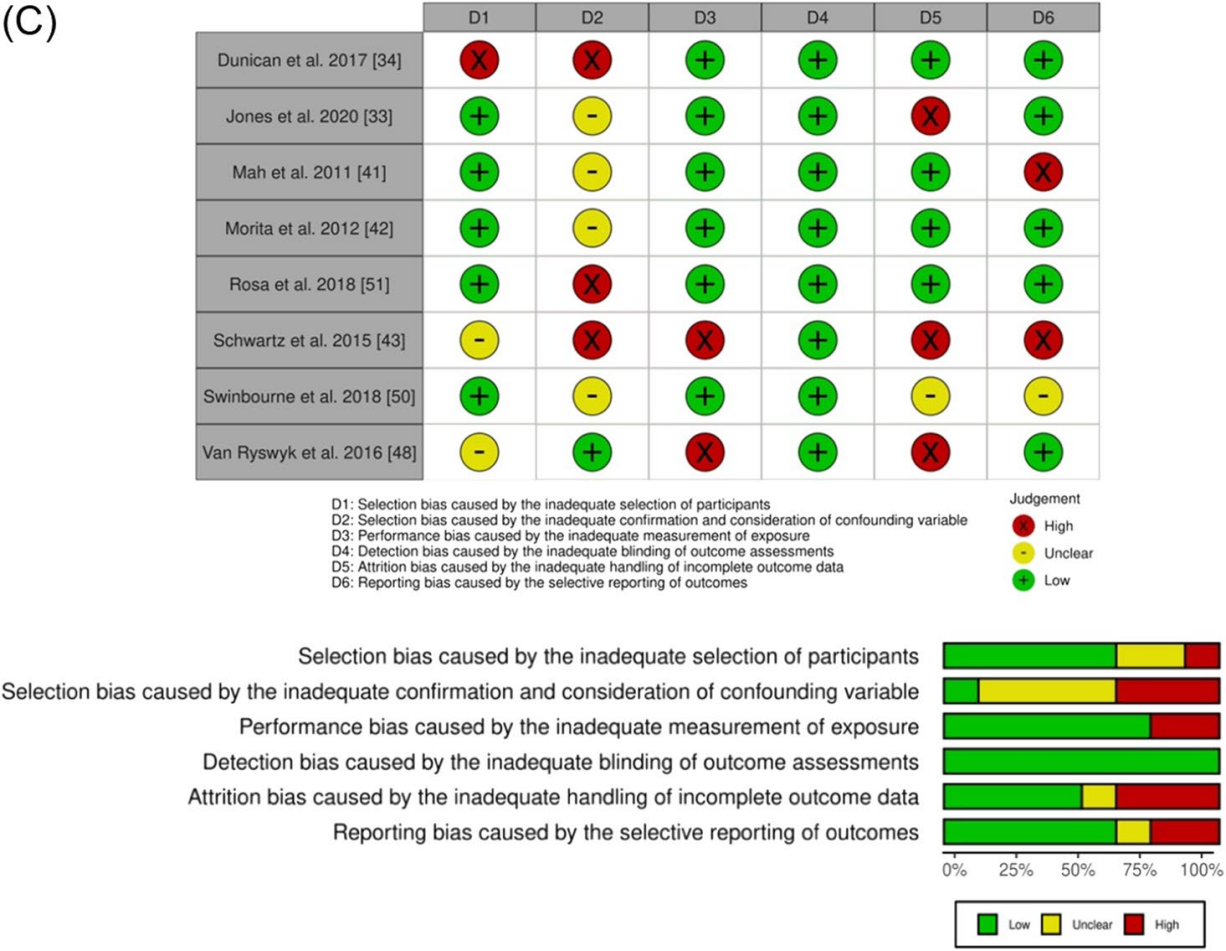
Risk-of-bias judgments by **A** RoB2 for crossover trials, **B** RoB2 for randomized controlled trials, and **C** RoBANS for non-randomized trials

The risk-of-bias analysis is summarized in Fig. 2. Figure 2A describes the analysis of crossover studies. Five studies were judged to be at a high risk of bias, and ten studies were considered with some concerns. Some possible biases were related to the carryover effects and outcome measurement (particularly the use of subjective tools), but the lack of pre-registration of intentions/methodology (which avoids the possibility of selecting the reported result) was particularly relevant for the overall results. Figure 2B describes the analysis of RCT. Two studies were judged to be at a high risk of bias due to the possible bias in all domains, except in domain 3 (missing outcome data). Figure 2C describes the analysis of non-RCT. Some potential biases were the lack of control over confounding variables and the lack of pre-registration of intentions/methodology.

### Effects of Sleep Interventions on Cognitive Performance

Twelve studies investigated the effect of sleep interventions on sleep and cognitive performance, of which one study explored sleep hygiene [48], four investigated naps [35, 42, 45, 49], one investigated naps combined with caffeine consumption [47], three investigated sleep extension [37, 41, 50], two investigated the removal of electronic devices [32, 34], and one investigated an artificial light intervention [51]. Only one study [48] examined the impact of sleep hygiene for six weeks and did not find any improvement on vigilance and attention, as measured by the Psychomotor Vigilance Task.

Regarding napping, despite differences in study design, a positive effect was found for short (20–40 min) and long naps (90–120 min) on attention, simple reaction time, multiple choice reaction time, juggling performance, mental rotation test, and lower reaction test [35, 42, 45, 49]. After a night of partial sleep deprivation and a typical night, Romdhani et al. [47] examined the effect of napping and caffeine or placebo and found that two-choice reaction time was improved, regardless of caffeine ingestion or sleep deprivation. For simple reaction time, performance was only enhanced after caffeine ingestion, regardless of sleep deprivation or napping.

Regarding sleep extension interventions, the results showed that increasing sleep duration positively impacts psychomotor vigilance task and reaction time [37, 41, 50].

Following the removal of electronics, Dunican et al. [32] and Jones et al. [34] did not observe any effects on sleep or cognitive performance (the Cogstate research tool and 5-min psychomotor vigilance task). Rosa et al. [51] examined the effect of artificial bright light on the sleep/wake cycle and psychomotor vigilance task performance. They observed an improvement in mean reaction time and a delay in the sleep/wake cycle, although sleep duration was significantly reduced.

In summary, naps, sleep extension, and light therapy demonstrated positive results on cognitive performance [35, 37, 41, 42, 45, 47, 49–51]. In contrast, removing electronic devices and sleep hygiene revealed no effects [32, 34, 48]. Data from these studies are summarized in Table 1.

### Effects of Sleep Interventions on Physical Performance

Sixteen studies investigated the effect of sleep interventions on sleep and physical performance, of which eight investigated the effect of napping [35, 36, 39, 40, 44, 45, 49, 52], one investigated naps combined with caffeine [46], three examined the impact of sleep extension [37, 41, 43], one investigated the effect of removing electronic devices [32], one investigated the effect of light intervention [38], one investigated the effect of mindfulness [33], and one investigated the effect of combining sleep hygiene and mindfulness [53].

Boukhris et al. [35], Romdhani et al. [45], and Nishida et al. [44] investigated the effects of short (20–40 min) and long naps (60–90 min) on maximal strength (knee extensors maximal voluntary isometric contraction), anaerobic capacity (running-based anaerobic sprint test and 5-min shuttle run test), and handball performance. The results showed a positive effect of naps, although Boukhris et al. [35] observed greater effects with a longer nap, and Nishida et al. [44] found positive effects following a 60-min nap in individuals with poor sleep quality. Boukhris et al. [39] and Hsouna et al. [40] examined the effect of a 40-min nap compared with no nap on the 5-min shuttle run test. They found a positive impact in total and best distance, despite no differences in fatigue index.

Despite the different contexts, Petit el al. [36] and Daaloul et al. [49] found no effects of napping when examining the impact of a short nap (20–30 min) on the Wingate test, squat jump, countermovement jump (CMJ), and karate-specific test. In a study divided into two experiments, Suppiah et al. [52] showed that napping negatively impacted sprint performance in track and field athletes. However, no effect was observed on shooting performance in shooters. Romdhani et al. [46] showed that a 20-min nap combined with caffeine improved running-based anaerobic sprint test (RAST) performance. In addition, caffeine without nap and a 20-min nap with a placebo improved maximum power.

Regarding sleep extension, increasing sleep duration improved basketball and tennis performance (timed 282 feet sprint, free throw, three-point shooting accuracy, and serving accuracy) and endurance capacity [37, 41, 43]. With respect to removing electronic devices and light intervention studies, Dunican et al. [32] did not show any effect of removing the electronic devices on the single leg three hop test, while Zhao et al. [38] found that 14 days of red-light treatment at night increased sleep quality and endurance performance (Cooper 12-minute run test). Jones et al. [33] showed that an 8-week mindfulness-based stress reduction program improved rowing performance (6000-m ergometer test). In contrast, Lever et al. [53] showed no improvement in tennis match performance (games won or lost) after combining mindfulness and sleep hygiene education.

In summary, napping interventions had conflicting results since five studies showed a positive effect [35, 39, 40, 44, 45], one showed positive results but was combined with caffeine [46], three showed no effect [36, 49, 52], and one a negative effect [52]. All sleep extension studies demonstrated improvements in physical performance [37, 41, 43], as did light [38] and mindfulness interventions [33]. Lastly, removing electronic devices [32] or combining mindfulness with sleep hygiene [53] did not affect physical performance measures. The main effects are summarized in Table 1.

### Effects of Sleep Interventions on Recovery

Five studies investigated the effect of sleep interventions on sleep quality, sleep duration, and recovery, of which one study investigated the effect of an acute sleep hygiene intervention [54], two investigated the effect of naps [39, 44], one investigated the effect of cold water immersion [55], and one investigated the impact of combining three strategies [56].

Concerning naps, Boukhris et al. [39] studied the effect of a 40-min nap on levels of muscle damage and inflammatory responses and found positive effects. On the other hand, Nishida et al. [44] compared the effect of short (20 min) and long naps (60 min) compared with no naps on HRV and did not find any difference between experiments. Chauvineau et al. [55] compared the effect of whole-body and partial-body cold water immersion with the control condition (i.e., sitting 10 min in a controlled environment) on HRV, wellness (Hooper index), creatine kinase, CMJ, and maximal isometric strength 24 h and 48 h post-intervention and found no differences between conditions. In regard to sleep hygiene, Fullagar et al. [54] demonstrated that a sleep hygiene intervention following a late-night soccer match had no effect on physical recovery (Yo–Yo intermittent recovery test—level 2 and CMJ 12 h and 36 h post-match, submaximal interval-based running test 18 h and 42 h post-match) or in blood markers of muscle damage and inflammation (C-reactive protein, creatine kinase, and urea 10 h, 20 h, 34 h, and 44 h post-match). Duffield et al. [56] combined sleep hygiene, full-body compression, and cold water immersion after and between two sessions of on-court tennis on the same day. They found a reduction in muscle and joint soreness the next morning, and large effect sizes, despite no significant differences, in reducing fatigue and increasing total sleep time, but no differences in vigor or sleep efficiency.

In summary, cold water immersion [55] and sleep hygiene [54] had no impact on performance recovery, muscle damage, or inflammation. Naps positively impacted muscle damage and inflammation [39], despite no effect on HRV [44]. Combining sleep hygiene, cold water immersion, and full-body compression positively affected muscle and joint soreness [56].

## Discussion

Sleep is widely recognized as one of the foundations for optimal health, well-being, and performance for athletes [10, 13, 57]. This systematic review synthesized the evidence regarding sleep interventions aimed at improving sleep, and whether this improvement would influence performance and/or recovery. Twenty-five studies with different sports and sleep interventions were analyzed. The findings reinforced that napping and sleep extension were the most promising sleep interventions for improved sleep and subsequent physical and/or cognitive performance.

All the included studies that implemented sleep extension showed a positive impact on performance outcomes (e.g., reaction time or sport-specific performance). The results showed that extending sleep duration by 46–113 min (e.g., increase of 11–27%) over 3–49 nights in athletes that habitually slept ~ 7 h per night may be a logical recommendation for future sleep extension programs [37, 41, 43, 50]. To achieve such sleep duration, studies have reported that athletes needed to extend their usual time in bed to 9–10 h [37, 41, 43, 50]. In addition, the individual characteristics of the athlete's sleep habits should be considered before implementing any sleep intervention, as it is important to adapt to individual needs [24]. Lastella et al. [9] demonstrated, in a sample of 124 participants, that elite athletes obtained well below 8 h of sleep per night (6.8 ± 1.1 h), which may not be enough to maximize performance. For example, Sargent et al. [8] revealed that elite athletes reported needing approximately 8.3 h of sleep to feel rested. Despite this self-reported sleep need, only 3% of athletes obtained their required sleep amount. It is plausible that athletes who fall short of their sleep requirements are likely to benefit from a sleep extension intervention.

Napping was the most representative sleep intervention of the included studies [35, 36, 39, 40, 42, 44, 45, 49, 52]. Napping is a strategy that provides an opportunity to supplement the night-time sleep period, offering an alternative for athletes to increase sleep duration. In most of the studies analyzed, the results revealed a positive impact of napping on cognitive performance [35, 42, 45, 49]. However, some mixed results were reported for physical performance [35, 36, 45, 49, 52] and recovery [39, 44] outcomes. During nights of partial sleep restriction (e.g., 4 h of sleep), decreases in general performance outcomes such as muscle power or psychomotor vigilance were observed [4]. The included studies showed that napping could restore performance to baseline levels [45, 49]. With regard to nap duration, the improvements in physical and cognitive performance were greater in naps of longer duration (e.g., 90 min) compared with shorter durations (e.g., 40 min) after a normal night [35]. However, following a night of partial sleep restriction, a short nap (20 min) was as effective as a long nap (90 min) in restoring performance to baseline levels (normal sleep night) [45]. There are still questions about the effects of daytime naps on night-time sleep variables (e.g., latency), especially naps of longer durations [36, 58]. Further, the notion of sleep inertia (e.g., feeling of grogginess upon wakening) needs to be considered when napping is implemented. For example, the longer the nap the higher the chance of experiencing sleep inertia [59]. A period of at least 30 min should be allowed after a nap to avoid the detrimental effects of sleep inertia on physical or cognitive performance [60], particularly for naps longer than 90 min. Based on the results of the included studies, it is unlikely that naps negatively affect performance outcomes in athletes. For all included studies, only one showed that napping decreased sprint performance in track-and-field athletes. However, the study did not reveal any effects on other sprint variables or shooting performance in pistol and rifle shooters [52].

The results of sleep hygiene interventions showed no effect on performance recovery, blood markers of damage (creatine kinase) and inflammation (C-reactive protein), or cognitive performance [48, 54]. Sleep hygiene currently refers to a list of behaviors, environmental conditions, and other sleep-related factors believed to promote improvements in sleep duration and quality [61]. Van Ryswyk et al. [48], contrasting with other studies that applied sleep hygiene with success [62, 63], concluded that a sleep hygiene protocol did not improve sleep quality and/or duration. Fullagar et al. [54] found that a sleep hygiene protocol resulted in a significant improvement in sleep duration after a late night soccer match compared to no sleep hygiene (6:09 ± 0:43 h vs 4:30 ± 0:27 h; *P* < 0.05), but no differences in recovery were observed between conditions. A possible explanation could be that even with an improvement in sleep duration, this improvement may not be enough, as it was still far from the 7 h of the minimum recommended sleep duration [5].

In studies that investigated the removal of electronic devices, the results showed no impact on sleep and/or athletic/cognitive performance [32, 34]. The main objective of this strategy is to reduce the exposure to the artificial light emitted by screens, especially before bedtime, reducing the decline in sleep quality and disturbance in the biorhythms [64]. Biorhythms are explained by the oscillation levels of endogenous hormones, like melatonin or cortisol, that regulate the sleep–wake cycle [65]. Despite this rationale, the results showed that the removal of electronic devices did not result in any change in sleep [32, 34]. Based on these results, it is unlikely that the removal of electronic devices should be on the “first line” of sleep interventions, because the difficulty of adherence by the athletes should also be considered. In addition, using electronic devices up to one hour before bedtime with the “night shift” mode (short-wavelength light limitation) significantly reduced melatonin suppression when compared with blue-light exposure (10 ± 2.7% vs 41 ± 4.1%) [66]. Furthermore, if devices were used with low light levels, the suppression of 10% may have been even smaller. It appears that using electronic devices in these circumstances, within reasonable limits, may not be problematic for athletes’ sleep. Moreover, it is still unclear whether ~ 10% of melatonin suppression induces circadian disruption and whether the type of activities (e.g., social media or gaming) can promote cognitive arousal.

Concerning light interventions, two studies investigated the effect of phototherapy but with different objectives. Zhao et al. [38] studied the effect of whole-body red-light phototherapy on sleep quality and endurance performance. At the same time, Rosa et al. [51] investigated the effect of bright-light therapy on sleep/wake cycle and reaction time. It is known that light is the most powerful circadian synchronizer for humans, which begins with its reception in the eyes and finishes in the pineal gland, which produces melatonin, a neurohormone essential for the functioning of the body-clock [67]. Zhao et al. [38] applied red-light therapy, which, unlike bright light, appears to have no impact on melatonin suppression [68] and may improve recovery [69, 70]. Despite increased sleep quality and melatonin levels after red-light therapy, it is important to interpret these results cautiously. The study used an inappropriate tool (the Pittsburgh Sleep Quality Index) to examine sleep quality over a short period and only analyzed melatonin levels upon waking. The authors also found a higher increase in running distance (Cooper 12-min run test) in the red-light treatment group (12.8%) compared to the control group (5.5%; *P* < 0.05). As the method used to examine sleep over a short period was unreliable and did not measure any exercise recovery outcome, these conclusions must be interpreted cautiously. In contrast, Rosa et al. [51] showed that bright-light therapy in the evening delayed the sleep/wake cycle and improved reaction time, promoting higher levels of alertness in a period when the athletes would usually be preparing for sleep. Further studies are needed to confirm these findings, considering that bright-light therapy can manipulate the body-clock to compete at night when alertness is usually already starting to drop.

The rationale for the possible positive effects of cold water immersion on sleep is that cold water immersion could accelerate the declining core body temperature and the reactivation of parasympathetic activity after exercise [71, 72]. Despite this, Chavineau et al. [55] did not find any impact on sleep architecture, muscle damage, core temperature, or fatigue, while Duffield et al. [56], combining cold water immersion with sleep hygiene and full-body compression, showed a positive effect on muscle/joint soreness and sleep duration (large effect sizes though non-significant results). However, Duffield et al.’s study [56] applied sleep hygiene and compression garments in addition to cold water immersion, and this could explain the differences between the results of the studies.

Finally, the studies that investigated the effect of mindfulness combined with sleep hygiene or mindfulness alone showed promising results. For example, Lever et al. [53] showed that combining sleep hygiene with mindfulness during a tennis tournament improved sleep duration, compared with a control group without any intervention (7.1 h vs 7.7 h). Despite an increase in sleep duration, no performance or sleep quality improvements were observed. Since this study only examined general performance that may be influenced by several factors (games won or lost), the impact of this type of intervention needs to be examined in detail in future studies. Jones et al. [33] tested a mindfulness intervention and showed no effect on sleep duration but did find improvements in sleep quality, mindfulness tendency (Five Facet Mindfulness Questionnaire) and rowing performance (6000-m ergometer test). It is plausible that mindfulness can improve sleep quality and/or duration; it could also positively impact physical/cognitive performance, as seen in sleep extension studies. However, it remains to be clarified whether the increase in mindfulness directly benefits athletic performance via attentional strategy, because mindfulness training appears to improve performance in precision sports such as shooting and dart throwing. Still, few controlled experimental studies have investigated the effects in non-precision sports [73].

In summary, this systematic review updated the knowledge about several sleep interventions' effects on improving sleep and subsequent performance in athletes. In 2017, Bonnar et al. [24] conducted a systematic review with a similar aim to the present review. Since then, many studies have been published and our understanding about the phenomena has improved. In the present review, we were able to include 15 more studies that brought new interventions, supporting some of the conclusions and showing different directions in other topics, compared to those of Bonnar et al. [24]. The effectiveness of sleep extension programs was reinforced with two new studies that showed, once again, the positive effects on sleep and subsequent performance. Napping was the most studied intervention since Bonnar et al.'s [24] study. The present review includes eight new studies that gave a different perspective, identifying positive effects on cognitive performance, despite mixed results on physical performance and recovery, while Bonnar et al. [24] only had two studies included with very different protocols, which did not allow for an in-depth discussion. The amount of studies examining sleep hygiene remained the same, considering that Duffield et al.’s study [56] also included compression and cold water immersion and Lever et al. [53] included mindfulness, suggesting that more research is warranted. New interventions, such as mindfulness, removing electronic devices at night or sleep/wake cycle manipulation through bright-light exposure are included in this review. Bonnar et al.’s review pointed out the lack of studies that evaluated cognitive techniques to improve sleep and subsequent performance. The negative impact of increasing arousal on the pre-competition night due to stress and anxiety, a common situation in the sports field, is known [74]. Although we are far from a definitive conclusion about the effect of cognitive interventions, such as mindfulness, researchers are beginning to be aware of the importance of studying this topic. Our review reinforces the importance of this line of research that could attenuate the detrimental effects of cognitive arousal on pre-competition night and improve sleep.

**Fig. 3:**
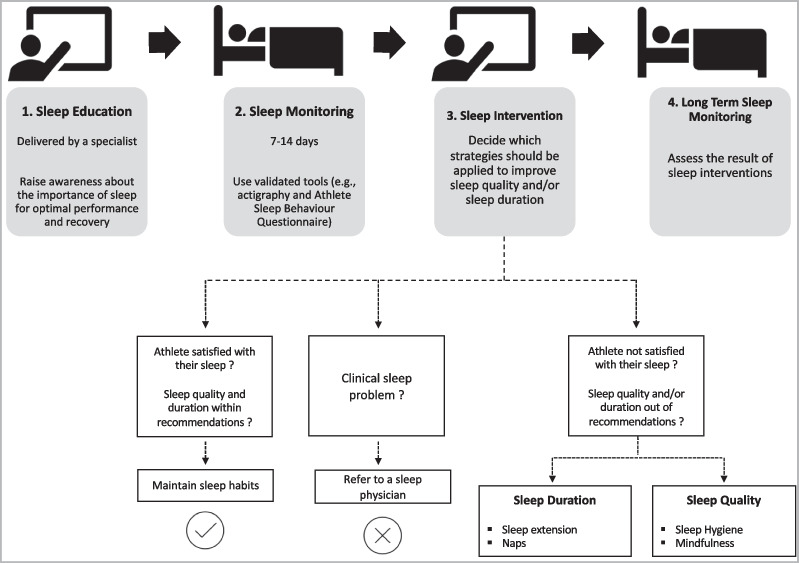
An example of how to implement a sleep intervention aimed at improving sleep and subsequent performance/recovery

Before implementing any strategy to improve sleep in athletes, there is some information that should be considered (Fig. 3). The first step should to provide a sleep education session, conducted by a specialist. The goal will be to raise awareness among athletes and coaches about the importance of sleep for optimal performance/recovery and explain the different strategies that could be used [10, 24]. After that, the habitual sleep/wake patterns should be identified [10]. This should be done with reliable and validated tools (e.g., actigraphy) for 7 to 14 days and apply validated questionnaires in athletes (e.g., Athlete Sleep Behaviour Questionnaire) [74]. This will facilitate an individualized approach to meet individual needs and identify the athletes with clinical sleep issues (e.g., insomnia) who should be referred to a sleep physician. Some caution should be taken with sleep monitors, as some athletes may be concerned about sleep monitor data, which may increase anxiety and result in worse sleep [10, 74].

To implement a sleep extension program, it is necessary to consider whether the athlete is obtaining adequate sleep for their needs [22]. If the athlete feels the need to sleep more, the recommendations based on included articles could be applied, although with some caution, as none of the included studies showed a low risk of bias. Furthermore, it is important to consider that to increase sleep duration based on the recommendations of this review, athletes may need to extend their usual time in bed to 9–10 h [37, 41, 43, 50]. In cases of athletes who are satisfied with the amount of sleep that they usually get, the possibility of increasing wakefulness in bed should be considered before trying to extend sleep duration. Napping is an alternative strategy to supplement insufficient night-time sleep, but can also be beneficial for those who only want a boost in alertness during the day [10, 60].

Sleep hygiene is a set of behaviors that should be implemented, although its effectiveness may be limited when used alone [48, 53, 56]. Implementing mindfulness is a long-term strategy because it needs to be learned. Preliminary evidence showed the long-term effect of mindfulness (5 to 12 months) on sleep [75], although Jones et al. [33] showed positive results on sleep quality within a 9-week period (one session per week). More research is needed to allow more solid recommendations. Motivation is also an important variable when a behavioral change is necessary, as in the case of sleep hygiene and mindfulness, and should be considered. Future research should investigate the impact of this variable on the effectiveness of behavioral interventions.

Long-term sleep monitoring should be conducted (sleep logs or validated devices) to assess whether interventions resulted in better sleep [10].

### Limitations

The current systematic review has limitations which should be acknowledged. First, the strengths of our conclusions are limited, since none of the included studies presented a low risk of bias. This is in line with a recent expert consensus statement about athletes' sleep studies, warning about the poor quality of the evidence, and the need to use more consistent, reliable, and valid research methods in athletes’ sleep studies [10]. To reduce the risk of bias, future studies should: design randomized controlled or crossover trials; identify the chronotype, due to possible bias in performance measures; explain in detail the randomization process; monitor a baseline sleep period (1–2 weeks) without any intervention; consider the carry over effect of the interventions, in the case of crossover trials; use objective and/or subjective validated tools for monitoring sleep; use objective performance measures; report missing data; and register the protocol before conducting the study. Second, there was high heterogeneity between the type of sleep interventions, type of sport, performance tests used, and level of the athletes. Lastly, there is a risk of language bias because we only considered studies written in English.

## Conclusion

Based on the results of this systematic review, increasing sleep duration through naps or night-time sleep may positively impact physical and/or cognitive performance. If athletes habitually obtain ~ 7 h of sleep per night, a general recommendation may be to increase sleep duration up to 2 h over 3–49 nights. Also, supplementing sleep during the day with a nap (20–90 min) can be implemented when necessary. In addition to improving the sleep duration, naps can improve performance outcomes after a regular night and restore performance decrements to baseline levels after a night with partial sleep restriction. For strategies such as sleep hygiene, mindfulness, or limiting the use of electronic devices before bedtime, it is plausible that such interventions can positively impact performance outcomes if they can improve sleep quality and/or duration. Strategies with light exposure may be an option to manipulate the biological clock and increase the alertness of the athletes in the moments when this starts to fall (e.g., at night). However, more studies are needed to confirm these findings. Future research on this topic should use more reliable and valid research methods to increase the quality of evidence so that more solid conclusions can be drawn. In addition, studies that explore the effect of 1–3 h of sleep restriction on physical and cognitive performance may be interesting, as this is the most common situation detected in the athletes’ context.

## Data Availability

Not applicable.
